# The Average Time Gap Between CA-125 Tumor Marker Elevation and Confirmation of Recurrence in Epithelial Ovarian Cancer Patients at Princess Noorah Oncology Center, Jeddah, Saudi Arabia

**DOI:** 10.7759/cureus.9518

**Published:** 2020-08-02

**Authors:** Ali Moharrag, Faisal Yonbawi, Hussam H Bashawieh, Ahmed Basabrain, Hatim M Al-Jifree

**Affiliations:** 1 Medicine, College of Medicine, King Saud Bin Abdulaziz University for Health Sciences, Jeddah, SAU; 2 Medicine and Surgery, King Saud Bin Abdulaziz University for Health Sciences, Jeddah, SAU; 3 Oncology, Ministry of National Guard Health Affairs, Jeddah, SAU; 4 Oncology, King Abdullah International Medical Research Center, Jeddah, SAU

**Keywords:** ca-125, epithelial ovarian cancer, ovarian cancer, tumor marker ca-125

## Abstract

The monitoring of the tumor marker cancer antigen 125 (CA-125) is commonly used as a part of epithelial ovarian cancer monitoring for recurrence. This study seeks to calculate the average time between CA-125 elevation above 35 IU/mL and evidence of recurrence through any currently accepted modality (positive clinical findings, biopsy, imaging, or PET [positron emission tomography] findings) in a patient population in Jeddah, Saudi Arabia.

We studied patients who were diagnosed between January 2006 and December 2016, underwent successful primary therapy, and were then followed up at Princess Noorah Oncology Center, King Abdulaziz Medical City, Jeddah, Saudi Arabia. We adopted a cross-sectional chart review study design. We used inclusive (consecutive) sampling.

A total of 13 patients were included, of whom 76.9% (10 patients) developed CA-125 elevations above 35 IU/mL prior to the confirmation of recurrence. If all 13 patients are included in the mean average calculation, the mean average time elapsed between CA-125 elevation and confirmation of recurrence was 161.5 days (standard deviation ± 230.6). If only the 10 patients who did exhibit a CA-125 elevation above 35 IU/mL were included, the mean average was 210 days (standard deviation ± 244.2).

## Introduction

Epithelial ovarian cancer is the fourth most lethal cancer in women. It primarily affects women over 50 years of age. Its types include serous, endometrioid, clear cell, mucinous, Brenner (transitional cell), and mixed epithelial tumors [1]. The recurrence rate is 60-70% in patients with low-level residual disease, but it climbs to 80-85% with high residual disease [2]. Tumor markers are biochemical substances that may be detected in cancer patients [3]. The best tumor marker to detect epithelial ovarian cancer is cancer antigen-125 (CA-125) [4]. A murine monoclonal antibody for CA-125, first developed by Bast et al. in 1983, made it possible to track the level of CA-125 in serum. Bast et al. indicated the top normal value for CA-125 to be 35 IU/mL [5]. Recurrence is indicated by monitoring certain patterns of CA-125 elevation in patients who have undergone primary treatment for epithelial ovarian cancer. There are no studies (to our knowledge) assessing the time gap between CA-125 tumor marker elevation and confirmation of epithelial ovarian cancer recurrence in Saudi Arabia.

Values double the top normal value are the classical indicator of recurrence. A preliminary study by Wilder et al. suggests that three consecutive serum CA-125 value increases within the normal range predicted recurrence within 4 to 24 months [6]. Similar findings are observed by Santillan et al. who additionally proposed that increases in the serum CA-125 level by 5 U/mL and 10 U/mL are also highly predictive of disease recurrence [7]. The poor prognostic outlook of patients with recurrent epithelial ovarian cancer necessitates an intensive follow-up program. A study states that serum CA-125, physical examination, and imaging examinations are the necessary elements to be used for the follow-up of asymptomatic patients. An advantage of CA-125 lies in its early detectability. Its elevation typically precedes clinical manifestations in 56-94% of patients with a mean average time of three to five months [8]. However, there is a considerable proportion of patients with recurrence who are negative for CA-125. Therefore, definitive confirmation of recurrence is dependent on radiological evidence [9]. A large, multicenter prospective study in the European Union questions the value of CA-125 surveillance post-primary therapy. While CA-125 is found to have given marker-positive patients a 4.8-month headstart, survival was not significantly improved. Furthermore, quality of life is found to have been impaired on average two months earlier in patients in CA-125 monitored patients [10].

We aimed to calculate the time gap between CA-125 simple elevation above 35 IU/mL and evidence of recurrence through any currently accepted modality (positive clinical findings, biopsy, imaging, or PET [positron emission tomography] findings) in a patient population where this time gap had not been investigated before (Jeddah, Saudi Arabia).

## Materials and methods

We studied epithelial ovarian cancer patients who underwent successful primary therapy at Princess Noorah Oncology Center, King Abdulaziz Medical City, Jeddah, Saudi Arabia, during the time period between January 2006 and December 2016. The study subjects were patients who received an initial diagnosis of epithelial ovarian cancer and were found to have an elevated serum CA-125 level at the time of diagnosis (>35 IU/mL). The patients experienced a full clinical and radiographic response to initial treatment with serum CA-125 returning within the normal parameters (<35 IU/mL) and then finally experienced recurrence confirmed through any modality (i.e. positive clinical, biopsy, imaging, or PET findings). We excluded all patients who did not satisfy all of these inclusion criteria. In total, 13 patients satisfied the criteria, with a mean average age at diagnosis of 51.8 years.

We adopted a cross-sectional chart review study design, with the data being extracted from physical and electronic medical records from the period between January 2006 and December 2016. We used inclusive (consecutive) sampling.

We used a data extraction sheet to collect information. The data extraction sheet included three parts: demographic information, medical information, and a CA-125 follow-up documentation sheet. We documented follow-up dates and CA125 values (in IU/mL) at each follow-up, including the date and value of elevation above 35 IU/mL. The date of confirmation of recurrence (determined by positive clinical, imaging, biopsy, or PET findings) was also documented.

To safeguard confidentiality, we serialized the data extraction sheets and kept them secure at all times. Identifiers (e.g. patient names, medical record numbers) were not collected. Ethical approval was obtained from the King Abdullah International Medical Research Center prior to the commencement of the study.

We deducted the date of CA-125 assay elevation above 35 IU/mL from the date of confirmation of epithelial ovarian cancer recurrence through any modality. After the time gap was calculated for each patient in the way described, we calculated the mean value and standard deviation (SD). A one-sample t-test was used to calculate the P-value in an attempt to investigate the statistical significance of our finding of the mean average time elapsed between CA-125 elevation and confirmation of recurrence in contrast to previous literature. We used the Statistical Package for the Social Sciences (SPSS) software (IBM Corp., Armonk, USA) for data handling and statistical analysis.

## Results

In total, 13 patients satisfied the inclusion criteria set forth in the Methods section. The data was analyzed in accordance with the methods described in that same section.

The patients had a mean average age at diagnosis of 51.8 years (Table 1). The majority of cases received the histopathological diagnosis of serous histopathology (91.7% of cases). A minority of cases received the histopathological diagnosis of poorly differentiated histopathology (8.3%). Of the 13 patients, 91.7% (12 patients) of patients underwent some type of surgery as part of their definitive treatment protocol and 8.3% (1 patient) did not and received chemotherapy alone. Among the patients who underwent surgery, 58.3% underwent optimal debulking (defined as the absence of any residual disease nodules larger than 10 mm across the longest dimension), 16.7% underwent sub-optimal debulking (defined as having at least one residual disease nodule larger than 10 mm across its longest dimension), and 25% underwent other surgical interventions.

**Table 1:** Demographic Data

Mean Age at Diagnosis	Standard Deviation	
51.77	17.57	
Histopathology Type	Frequency	Percentage
Serous	12	92.31
Poorly differentiated	1	7.69
Procedure Type	Frequency	Percentage
Optimal debulking	7	53.85
Suboptimal debulking	2	15.38
Other	3	23.08
Chemotherapy Type	Frequency	Percentage
Taxane and carboplatin	4	30.77
Carboplatin single agent	3	23.08
Ribosomal doxorubicin and carboplatin	2	15.38
Taxane	1	7.69
Other	1	7.69
No chemotherapy	2	15.38

**Figure 1:**
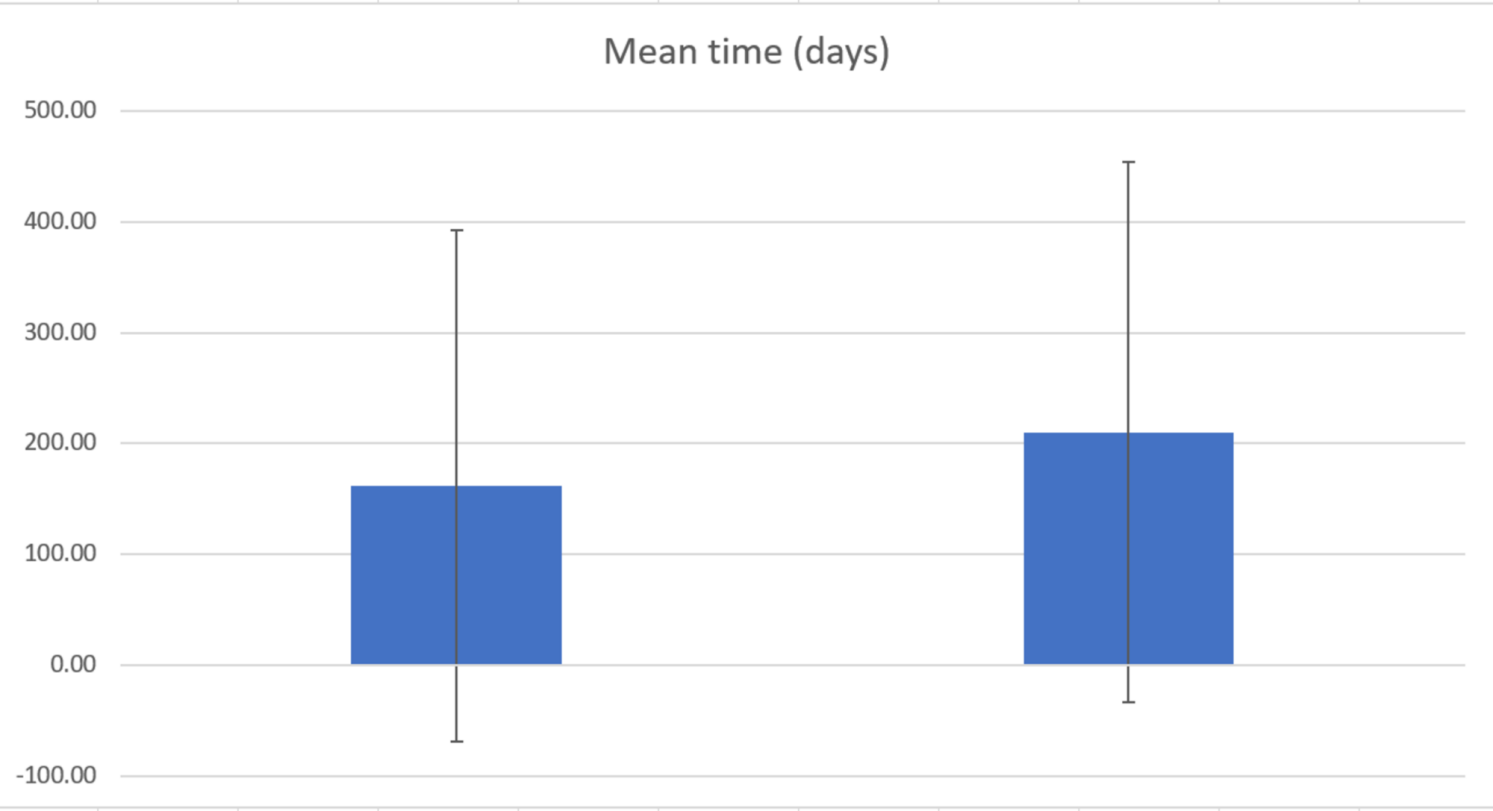
Mean Time (Day)

Out of 13 patients, 76.9% (10 patients) developed CA-125 elevations above 35 IU/mL prior to the confirmation of recurrence. If all 13 patients are included in the mean average calculation, the mean average time elapsed between CA-125 elevation and confirmation of recurrence was 161.5 days (SD ± 230.6) (Figure 1). If only the 10 patients who did exhibit a CA-125 elevation above 35 IU/mL were included, the mean average was 210 days (SD ± 244.2).

## Discussion

In a clinical review, Gadducci and Cosio found that repeated CA-125 assays during follow-up after initial remission precede clinical manifestations in 56-94% of patients. The mean average time between CA-125 elevation and confirmation of recurrence was three to five months [8]. Rustin et al., one of the largest prospective cohort studies conducted on the role of CA-125 elevation in the detection of recurrent ovarian cancer, found that the difference between CA-125 elevation and clinical confirmation of recurrence was 4.8 months [11]. Other studies reached a similar conclusion but emphasized that employing various imaging modalities in the detection of recurrence was found to yield an anticipatory advantage with regard to preventing the progression of symptoms. This conclusion was established based on finding an average of a 2.8-month interval between radiological evidence of recurrence and actual progression of symptoms [10]. Thus, the recommendation of initiating therapeutic protocols before the development of symptoms was made for the purpose of mitigating patients’ symptom-related anxiety and improving their quality of life [10]. In contrast to past studies, our study accepted imaging and biopsy findings as confirmatory measures for recurrence, in addition to clinical findings.

The majority of cases received the histopathological diagnosis of serous histopathology (91.7% of cases), which is the most common histopathological diagnosis according to past literature. The remaining 8.3% of cases had poorly differentiated carcinoma. Most management protocols (12 out of the total 13) included debulking surgery of some description and chemotherapy, which together constitute the most common definitive management in epithelial ovarian cancer patients.

Out of 13 patients, 76.9% (10 patients) developed CA-125 elevations above 35 IU/mL prior to the confirmation of recurrence. This finding is in line with a number of previous studies, which variously found this percentage to be between 56% and 94% [8]. When all 13 patients were included in the mean average calculation, the mean average time elapsed between CA-125 elevation and confirmation of recurrence was 161.5 days (SD ± 230.6). When only the 10 patients who did exhibit a CA-125 elevation above 35 IU/mL were included, the mean average was 210 days (SD ± 244.2).

The mean average time elapsed 161.5 days (SD ± 230.6) between elevation and confirmation of recurrence is longer than the gap found by Rustin et al. (144 days), perhaps reflecting that a purely clinical basis for the definition of recurrence was used by Rustin et al. [11]. Furthermore, our findings indicated a greater interval (mean: 161.5 days; SD ± 230.6) than the approximate 60 days between significant elevation of CA-125 and radiological detection of recurrence that was mentioned by Pignata et al. [10]. However, a one-sample t-test revealed this difference to be not statistically significant (P = 0.138). The mean average time gap that included only the 10 patients who did exhibit a CA-125 elevation above 35 IU/mL was even longer (mean 210 days, SD ± 244.2), but the difference was also not statistically significant (P = 0.084) from the time gap mentioned by Pignata et al [10].

The value of CA-125 surveillance post-primary therapy was called into question in the aforementioned study by Rustin et al. In that study, all patients’ CA-125 levels were monitored at regular intervals [11]. One group had their elevated values reported promptly to their physician, whereas the others did not [11]. The choice of whether or not to treat patients based solely on CA-125 elevation, and the choice of chemotherapeutic agents, was left at the physician’s discretion [11].

While Rustin et al. established that CA-125 did give marker-positive patients a 144-day time advantage, survival was not significantly improved [11]. Furthermore, quality of life was impaired on average two months earlier in patients who had their values reported [11]. The group therefore recommended that the patients be given the choice of whether to have their CA-125 levels monitored and be informed that, for most patients and under current therapy options, their prognosis is unlikely to be improved [11]. This suggests that with the current established therapeutic options, the 161.5-day gap we calculated is similarly unlikely to yield a significant improvement in survival and quality of life.

## Conclusions

We conclude that the mean average of time elapsed between the elevation of CA-125 and the confirmation of recurrence of epithelial ovarian cancer for all patients in our study was 161.5 days. This is not significantly different from the findings of previous research efforts.

The difference in mean time elapsed between all patients, including those who did not experience a second elevation of CA-125 to herald recurrence (mean: 161.5 days; SD ± 230.6) and the mean time elapsed when only those patients who did not experience an elevation before recurrence confirmation were included (mean 210: days; SD ± 244.2), seems to call into question the value of CA-125 surveillance for all patients, although it should be noted that there is no way for a clinician to know, without hindsight, which patients will experience a new elevation in the tumor marker and which will not, hence the utility of including all patients in our reported value of mean time difference.

We conclude that while the data gleaned in this study fall in line with previous literature, the sample size makes it unwise to conclude with certainty or to generalize these findings to the population at large. Furthermore, while the small difference between our findings and those found in previous literature suggests that, with current established therapeutic options, a 161.5-day gap is not likely to yield a significant improvement in survival and quality of life, we did not measure these outcomes in our patient population and therefore cannot make this statement with confidence.

Therefore, we recommend that a large, multicenter study focused on measuring survival and quality of life outcomes in patients with routinely reported CA-125 values as part of their routine surveillance monitoring versus those without be conducted in Saudi Arabia. We also recommend a prospective cohort study design. This is because prospective research is free from the limited data availability and uneven documentation that inevitably governs, and may even hinder, retrospective efforts.
